# The effectiveness of anterior unilateral bite turbos in correcting incisal plane cant and asymmetric overbite: a prospective clinical study

**DOI:** 10.1038/s41598-025-01035-w

**Published:** 2025-05-06

**Authors:** Viet Anh Nguyen, Thuy Anh Nguyen, Thi Bich Ngoc Doan

**Affiliations:** 1 Faculty of Dentistry, PHENIKAA University, Hanoi, Vietnam; 2 Private Practice, Viet Anh Orthodontic Clinic, Hanoi, Vietnam

**Keywords:** Incisal plane cant, Facial asymmetry, Anterior unilateral bite turbos, Corrective orthodontics, Minimally invasive dentistry, Dentistry, Occlusion, Orthodontics

## Abstract

This study aimed to evaluate the effectiveness of anterior unilateral bite turbos (AUBTs) in correcting incisal plane cant and asymmetric overbite in orthodontic patients. A prospective study was conducted on 40 orthodontic patients (4 males, 36 females) undergoing fixed appliance treatment in a private practice setting. Subjects presented with either primary or iatrogenic incisal plane cants and received AUBT placement on the upper canine or lateral incisor of the more pronounced overbite side. Digital photographic analysis was used to measure changes in cant parameters at baseline (T0), pre-AUBT placement (T1), and post-treatment (T2). Statistical analysis was performed using repeated measures ANOVA and Bonferroni post hoc tests. Results indicated that AUBTs significantly reduced incisal plane cant across all groups, with the primary group showing a greater overall improvement compared to the iatrogenic group. The lower incisal plane and gingival margin exhibited more significant cant correction than the upper ones. Patients adapted well to the appliances, with minimal debonding failures and no significant changes in temporomandibular function. This study supports the use of AUBTs as a minimally invasive, cost-effective alternative for incisal plane cant asymmetric overbite correction. Future research should explore long-term stability and comparative effectiveness with other cant correction modalities.

**Supplementary Information:**

The online version contains supplementary material available at 10.1038/s41598-025-01035-w.

## Introduction

Among anterior dental parameters such as incisal plane cant, mesiodistal angulation and torque of anterior teeth, and midline deviation, any discrepancy may negatively affect smile symmetry and dentofacial esthetics^1,2^. An incisal plane cant may arise from facial asymmetry or vertical discrepancies between the right and left quadrants of the dental arches^3^. Previous studies showed that a dental professional may perceive an incisal plane cant of 1.63° as significantly less esthetics^4,5^. Additionally, an incisal plane cant can be easier to detect than a midline shift with normal crown angulation^5^. The perception of an incisal plane cant is also influenced by other factors, such as the parallelism of the commissure line with the inter-pupillary line and the presence of a dental midline shift^6^. Recent efforts have been made to standardize its evaluation through the development and validation of the occlusal cant index^7^.

A dental occlusal cant generally expresses only in the anterior region, accompanied by an asymmetric overbite and normal posterior occlusal plane^8^. Several case reports have presented the successful management of incisal plane cant with intrusion arches, rollercoaster archwires, and multiloop edgewise archwires^9,10^. However, a skeletal occlusal cant with an asymmetric posterior occlusal plane and mandible deviation may require skeletal anchorage or orthognathic surgery to be corrected^11–13^.

Fixed anterior bite turbos have been demonstrated to be effective in reducing deep overbites, offering a simpler, more cost-effective approach with less reliance on patient compliance compared to removable bite planes^14^. Anterior bite turbos offer a larger amount of overbite reduction and a shorter duration compared to archwires with reverse curves of Spee and utility arches^15,16^. Based on these findings, the use of anterior unilateral bite turbos (AUBTs) has been proposed to correct asymmetric overbite and incisal plane cant in a case series^8^. However, this study’s limitations include a small sample size and the lack of quantification of the amount of incisal plane cant correction achieved.

Addressing incisal plane cant during fixed orthodontic treatment is essential to achieve optimal smile esthetics and facial symmetry. To our knowledge, there has been no original research investigating the effectiveness of orthodontic appliances for canting reduction in the literature. Therefore, this study aims to assess the effectiveness of AUBTs in correcting incisal plane cant and asymmetric overbite in vivo. The null hypothesis proposed that the use of AUBTs would not result in a statistically significant reduction in incisal plane cant in patients undergoing fixed orthodontic treatment.

## Materials and methods

### Subjects

This prospective study adheres to the Strengthening the Reporting of Observational Studies in Epidemiology guideline (STROBE) and the study protocol was approved by the PHENIKAA University Institutional Ethical Board Review (approval no PU-1-D-01, dated June 14th, 2022). All methods were performed in accordance with the relevant guidelines and regulations and adhered to the Declaration of Helsinki. The subjects were consecutive patients with an incisal plane cant and asymmetric overbite during fixed orthodontic treatment in private practice (Nam Tu Liem, Hanoi, Vietnam) from July 1st, 2022 to July 1st, 2024. All participants provided written informed consent. The incisal plane cant might be present before treatment or develop during treatment.

**Fig. 1:**
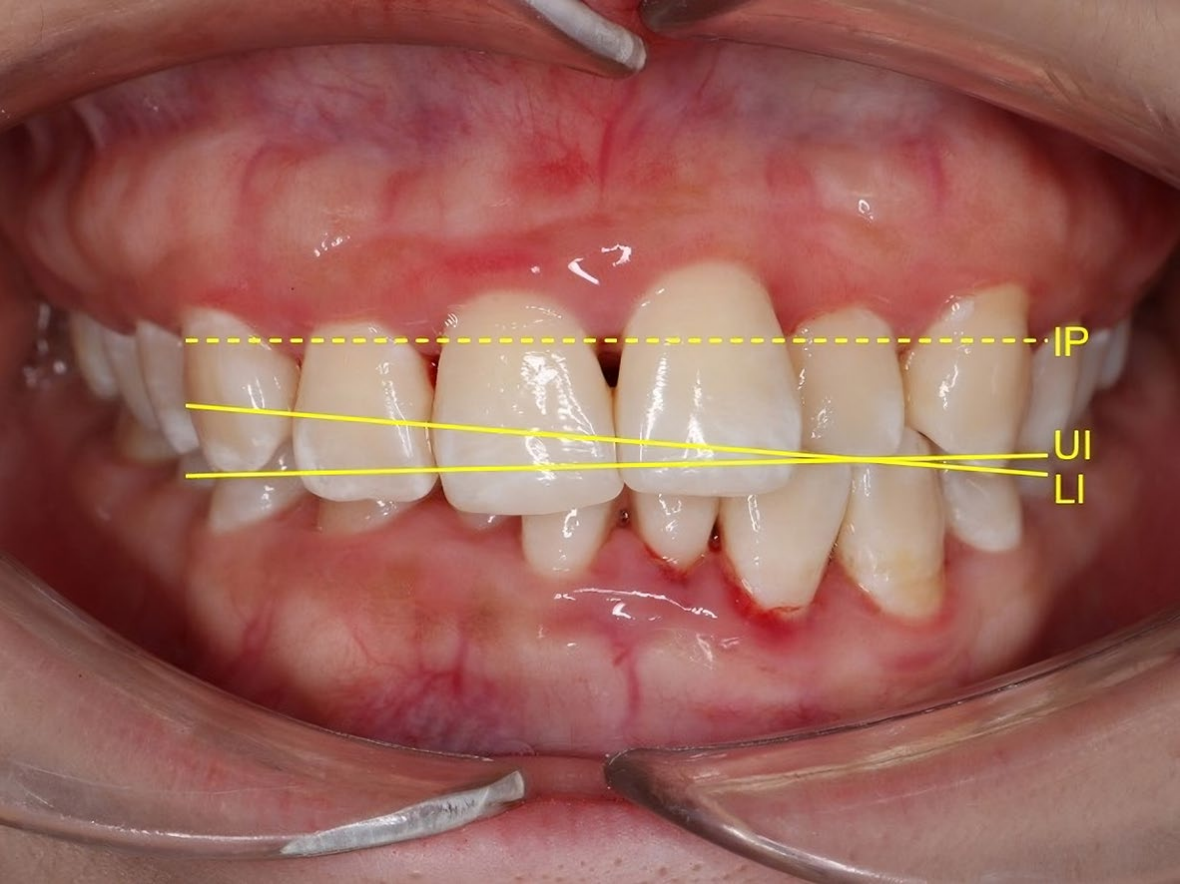
Pretreatment cant of the lower incisal plane (LI) relative to the upper incisal plane (UI) and asymmetric overbite. Ideally, both the upper incisal plane and the lower incisal plane should be parallel to the interpupillary line (IP).

The inclusion criteria were: (1) patients undergoing fixed orthodontic treatment, (2) presence of a cant of the lower incisal plane relative to the upper incisal plane (CLIUI) of at least 1.63°, as measured on standardized frontal intraoral photographs (Fig. 1), and (3) permanent dentition with fully erupted anterior teeth. The exclusion criteria included: (1) clinical signs or symptoms of temporomandibular disorders, including joint click, pain, or restricted mouth opening, (2) history of craniofacial trauma or surgery, and (3) presence of significant skeletal asymmetry or systemic conditions affecting craniofacial growth.

The sample was divided into primary and iatrogenic cant groups, in which the primary group included patients with a pre-existing incisal plane cant before treatment, while the iatrogenic group consisted of patients who developed an incisal plane cant during orthodontic treatment due to factors such as asymmetric mechanics, bracket positioning, or unwanted tooth movement.

Because no previous study assessed the effectiveness of cant correction, data from the study of Al-Zoubi et al.^17^ on the overbite-reducing effectiveness of fixed anterior bite turbo were used to calculate the sample size. The mean overbite correction from before to after treatment was 3.0° ± 1.5°, indicating a large effect size of 0.91. Based on the calculated sample size, analyzing 18 patients would provide 95% power to detect a significant cant correction difference from 1.63°. A 1 mm threshold has been established as the minimum occlusal cant deviation perceived as significantly less esthetic by dental professionals, as reported by Kokich et al.^5^ To translate this linear displacement into an angular measurement, the 1.63° threshold was derived using the arcsine function, based on a mean upper inter-canine width of 35.1 mm in Southern Asian females^18^.

### Bite turbo placement

**Fig. 2:**
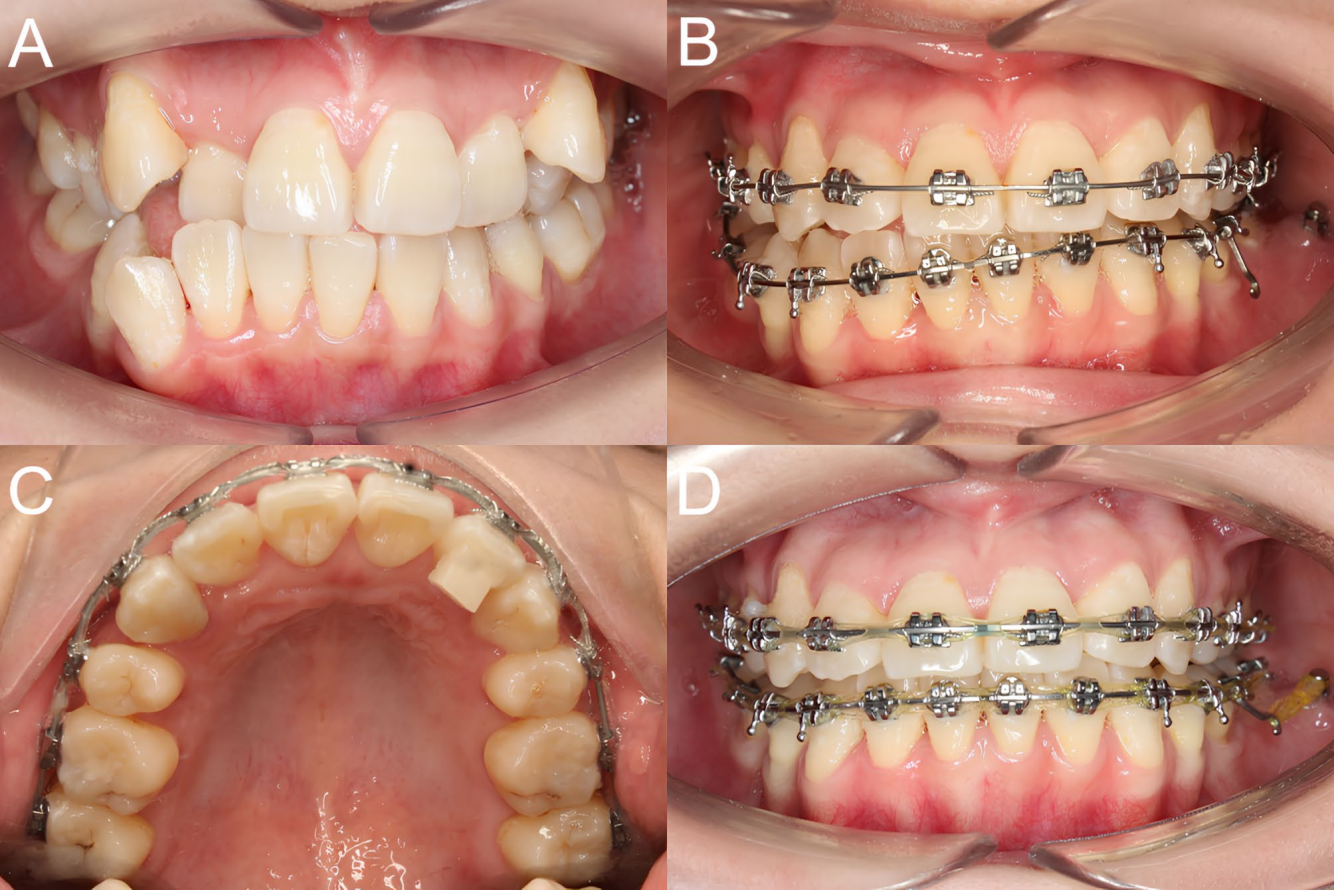
Anterior unilateral bite turbo for incisal plane cant and asymmetric overbite correction. (**A**) Pretreatment. (**B**) Incisal plane cant exacerbated due to unilateral distalization of the lower arch. (**C**) Bite turbo placement. (**D**) Three months after bite turbo placement.

The treatment was initiated by bonding 0.022” x .028” labial brackets (Trumpet, Medico, Korea) or 0.018” x .025” lingual brackets (ADB, Medico, Korea). Initial occlusal leveling was performed using flexible nickel-titanium archwires (NiTi, Medico, Korea) before progressing to stiff stainless steel continuous archwires engaged in both arches. The specific archwire dimensions were 0.019” x .025” stainless steel for labial brackets and 0.016” x .022” stainless steel for lingual brackets. For each patient, an AUBT was built up on the lingual surface of the upper canine on the side with a larger overbite, at a distance of about 2 mm from the incisal edge to create premature contact with the opposing lower teeth and establish a normal overbite on that side. Alternatively, an AUBT could be placed on the upper lateral incisor or both the upper canine and lateral incisor (Fig. 2). Generally, teeth selected for AUBT placement should have the highest vertical overlap with opposite lower teeth and healthy periodontium without attachment loss. A light-cured composite (Escom, Spident, Korea) was used to build up AUBTs. Open bites would develop on the contralateral side and posterior teeth due to premature contact, necessitating vertical triangle elastic application (3/16 inch, 3.5 ounces) to close the bite. No posterior bite raising would be placed to facilitate occlusal settling. In patients treated with lingual appliances, due to the restricted bonding area, AUBTs were bonded to the lingual tooth surface and partially to the bracket after sandblasting and universal primer application (Single Bond Universal, 3 M, Germany). AUBTs were generally maintained until fixed appliance removal. In some instances, AUBTS were removed earlier if they hindered occlusal settling during the finishing stage. A detailed description of the AUBT placement technique and clinical management has been presented in a case series^8^.

### Data acquisition

Digital photographs were taken with an EOS 750D camera (Nikon, Japan) capturing straight frontal views of patients’ faces and occlusions with mouth retractors, using a standardized setup to ensure consistency across all time points. Patients were seated with ear rods in place to minimize head movement, maintaining a natural head position with their Frankfort horizontal plane parallel to the floor. The camera was mounted on a tripod at a fixed distance and angle, ensuring consistent framing and perspective. Standardized lighting conditions were maintained throughout the image acquisition process. Three sets of photographs were taken for each patient: before treatment (T0), before AUBT placement (T1), and after treatment (T2). Angular measurements were obtained using virtual protractor software (Pissa Ruler, IO Stream, Vietnam). Intraoral scans were taken at each point to assist in determining lower incisal planes. The subjects were also monitored for signs and symptoms of temporomandibular disorders after AUBT placement.

**Fig. 3:**
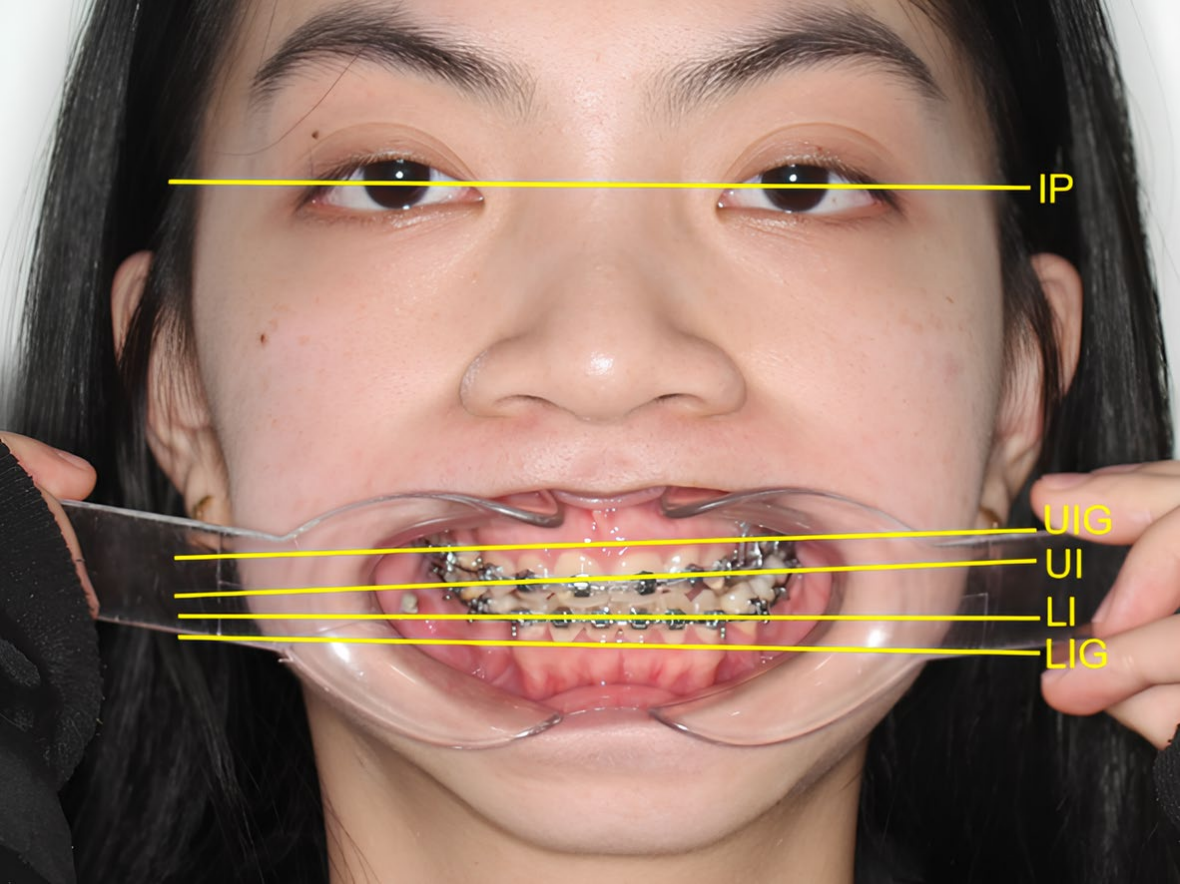
Straight frontal photograph of a patient’s face and occlusion with mouth retractors, showing incisal plane cant assessment using reference lines. *IP* interpupillary line, *UIG* upper incisal gingival margin, *UI* upper incisal plane, *LI* lower incisal plane, *LIG* lower incisal gingival margin.

Our analysis focused on six angular and linear variables, three measured at the incisal level and three at the gingival margin (Fig. 3). The incisal level measurements included the cant of the upper incisal plane to the inter-pupillary line (CUIIP), the cant of the lower incisal plane to the upper incisal plane (CLIUI), and the cant of the lower incisal plane to the inter-pupillary line (CLIIP). Similarly, the gingival margin level measurements were the cant of the upper incisal gingival margin to the inter-pupillary line (CUIGIP), the cant of the lower incisal gingival margin to the upper incisal gingival margin (CLIGUIG), and the cant of the lower incisal gingival margin to the inter-pupillary line (CLIGIP). To ensure that positive and negative changes would not negate each other, cants in the same direction as CLIUI at T1 were assigned positive values, while those in the opposite direction received negative values. Additionally, the changes in vertical dimension between T2 and T0 were recorded using the sella-nasion-to-mandibular plane (SNMP) angle measured on lateral cephalograms.

### Statistical analysis

All data analyses were conducted using SPSS software (version 23.0; IBM, Armonk, NY). The Shapiro-Wilk test was performed to assess the normality of the variables. Chi-square tests were performed to compare demographic characteristics between the primary and iatrogenic groups. Descriptive statistics (mean and standard deviation) were calculated for all parameters and their changes across different time points. Repeated measures ANOVA was used to evaluate significant differences in cant parameters over time, with Bonferroni post hoc tests applied for pairwise comparisons. Additionally, changes in cant parameters were compared between the primary and iatrogenic groups at T2–T1 and T2–T0. A statistical significance level of α = 0.05 was applied.

To assess operator measurement error, another operator remeasured all parameters of 10 random subjects. The second set of measurements was compared with the first set using intraclass correlation coefficients (ICC).

## Results

Forty treated subjects, 4 male and 36 female Asians were included in the study, including 15 primary and 25 iatrogenic cants. The mean age was 24.0 years at T0 and 25.9 years at T2. The average time from T0 to T1 was 14.3 months and the average AUBT duration was 5.9 months. In the iatrogenic group, asymmetric overbites and incisal plane cants resulted from leveling severely displaced teeth, asymmetric bracket height, unilateral distalization, asymmetric intermaxillary elastics application, and space closure. Unilateral distalization had the highest prevalence (48%) of iatrogenic incisal plane cant (Table 1). Chi-square tests revealed no significant differences in demographic characteristics between the primary and iatrogenic groups (*P* > 0.05) in terms of gender distribution, etiology, occlusion, overbite, vertical facial pattern, appliances, and extraction treatment.

**Table 1:** Description of the study group.

	Total	Primary	Iatrogenic	*P* value
Gender				0.296
Female	36	13	23	
Male	4	2	2	
Etiology	40	15	25	0.113
Displaced teeth			1	
Bracket height			2	
Unilateral distalization			12	
Intermaxillary elastics			6	
Space closure			4	
Occlusion				0.251
Class I	33	14	19	
Class II	3	1	2	
Class III	4	0	4	
Overbite				0.052
Reduced	7	0	7	
Normal	16	6	10	
Increased	17	9	8	
Vertical facial pattern				0.073
Brachyfacial	7	5	2	
Mesofacial	24	6	18	
Dolichofacial	9	4	5	
Appliances				0.170
Labial	29	9	20	
Lingual	11	6	5	
Extraction treatment				0.060
Extraction	21	5	16	
Non-extraction	19	10	9	
Age at T0 (y)	24.0 ± 5.3	26.1 ± 5.2	22.7 ± 5.0	0.908
Age at T2 (y)	25.9 ± 5.2	27.7 ± 5.1	24.8 ± 5.1	0.987
Time interval T1-T0 (mo)	14.3 ± 7.2	9.9 ± 5.4	16.9 ± 6.9	0.197
Time with turbos (mo)	5.9 ± 1.9	5.9 ± 1.9	5.9 ± 1.9	0.882

The inter-observer reliability was high, with ICCs for repeated measurements ranging from 0.903 to 0.918 at T0, 0.921 to 0.931 at T1, and 0.919 to 0.928 at T2. No changes in temporomandibular joints were observed after AUBT placement in any of the subjects. Although posterior bite raising was not used, difficulties in chewing were reported only during the first month, and patients adapted well to the AUBTs thereafter. Only 3 (7.5%) AUBTs failed in the whole sample due to debonding and breakage. The SNMP angle showed a non-significant increase of 0.30° (*P* = 0.221), 0.20° (*P* = 0.490), and 0.24° (*P* = 0.237) in the primary, iatrogenic, and whole sample groups, respectively.

Repeated measures ANOVA indicated statistically significant changes in cant parameters across different time points in all groups (*P* < 0.01). Changes in incisal-level and gingival-margin measurements followed similar trends. In the primary group, all parameters showed significant reductions at T2 compared to both T1 and T0, except for CUIIP and CUIGIP, which improved at T2 relative to T0 but did not reach statistical significance. No significant changes were observed between T0 and T1 (Table 2). In the iatrogenic group, cant parameters worsened significantly from T0 to T1 but showed a substantial reduction from T1 to T2, with no significant difference between T0 and T2 (Table 3). Additionally, CLIIP and CLIGIP demonstrated greater severity compared to CUIIP and CUIGIP at T0 and T1 in the primary group, as well as at T1 in the iatrogenic group.

**Table 2:** Changes in cant parameters over time in the primary group.

	T0 (°)	T1 (°)	T2 (°)	*P* Value
CUIIP	-1.10 ± 1.63^a, b^	-1.01 ± 1.24^a^	-0.13 ± 1.45^b^	0.004
CLIUI	4.40 ± 2.87^a^	3.27 ± 1.24^a^	0.25 ± 0.96^b^	< 0.001
CLIIP	3.29 ± 2.37^a^	2.26 ± 1.78^a^	0.13 ± 1.59^b^	0.001
CUIGIP	-1.50 ± 1.84^a, b^	-1.24 ± 1.28^a^	-0.61 ± 1.26^b^	0.012
CLIGUIG	6.01 ± 4.15^a^	3.88 ± 2.25^a^	1.27 ± 1.61^b^	< 0.001
CLIGIP	4.51 ± 3.36^a^	2.64 ± 2.38^a^	0.67 ± 1.65^b^	0.002

*CLIGIP* cant of the lower incisal gingival margin to the inter-pupillary line, *CLIIP* cant of the lower incisal plane to the inter-pupillary line, *CLIGUIG* cant of the lower incisal gingival margin to the upper incisal gingival margin, *CLIUI* cant of the lower incisal plane to the upper incisal plane, *CUIGIP* cant of the upper incisal gingival margin to the inter-pupillary line, *CUIIP* cant of the upper incisal plane to the inter-pupillary line. Values with different superscript lowercase letters (a, b) denote statistical significance between time points based on Bonferroni post hoc tests (*p* < 0.05).

**Table 3:** Changes in cant parameters over time in the iatrogenic group.

	T0 (°)	T1 (°)	T2 (°)	*P* Value
CUIIP	-0.26 ± 1.71^a^	-1.16 ± 1.76^b^	-0.49 ± 1.68^a^	0.001
CLIUI	0.28 ± 1.47^a^	2.66 ± 0.98^b^	0.44 ± 1.02^a^	< 0.001
CLIIP	0.02 ± 1.81^a^	1.51 ± 1.77^b^	-0.05 ± 1.81^a^	< 0.001
CUIGIP	-0.38 ± 1.71^a^	-1.32 ± 1.64^b^	-0.67 ± 1.58^a^	< 0.001
CLIGUIG	0.77 ± 1.95^a^	3.16 ± 1.63^b^	1.03 ± 1.79^a^	< 0.001
CLIGIP	0.28 ± 2.16^a^	1.84 ± 2.14^b^	0.36 ± 2.23^a^	< 0.001

*CLIGIP* cant of the lower incisal gingival margin to the inter-pupillary line, *CLIIP* cant of the lower incisal plane to the inter-pupillary line, *CLIGUIG* cant of the lower incisal gingival margin to the upper incisal gingival margin, *CLIUI* cant of the lower incisal plane to the upper incisal plane, *CUIGIP* cant of the upper incisal gingival margin to the inter-pupillary line, *CUIIP* cant of the upper incisal plane to the inter-pupillary line. Values with different superscript lowercase letters (a, b) denote statistical significance between time points based on Bonferroni post hoc tests (*p* < 0.05).

The comparison of cant corrections between the primary and iatrogenic groups from T1 to T2 revealed no statistically significant differences between the two groups for any parameter (*P* > 0.05) (Table 4). However, when comparing T2 to T0, the primary group exhibited significantly greater reductions across all measured parameters compared to the iatrogenic group (*P* < 0.05) (Table 5). Notably, the improvements in CLIIP and CLIGIP were more pronounced than those in CUIIP and CUIGIP.

**Table 4:** Changes in cant parameters between T2 and T1 in the total, primary, and iatrogenic groups.

	Total (°)	Primary (°)	Iatrogenic (°)	*P* Value
CUIIP	0.75 ± 0.87	0.88 ± 0.86	0.66 ± 0.88	0.441
CLIUI	-2.52 ± 1.33	-3.02 ± 1.46	-2.22 ± 1.18	0.068
CLIIP	-1.77 ± 1.53	-2.13 ± 1.88	-1.56 ± 1.28	0.258
CUIGIP	0.64 ± 0.84	0.63 ± 0.79	0.65 ± 0.88	0.944
CLIGUIG	-2.31 ± 1.40	-2.60 ± 1.59	-2.13 ± 1.27	0.304
CLIGIP	-1.66 ± 1.53	-1.97 ± 1.78	-1.48 ± 1.36	0.326

*CLIGIP* cant of the lower incisal gingival margin to the inter-pupillary line, *CLIIP* cant of the lower incisal plane to the inter-pupillary line, *CLIGUIG* cant of the lower incisal gingival margin to the upper incisal gingival margin, *CLIUI* cant of the lower incisal plane to the upper incisal plane, *CUIGIP* cant of the upper incisal gingival margin to the inter-pupillary line, *CUIIP* cant of the upper incisal plane to the inter-pupillary line.

**Table 5:** Changes in cant parameters between T2 and T0 in the total, primary, and iatrogenic groups.

	Total (°)	Primary (°)	Iatrogenic (°)	*P* Value
CUIIP	0.22 ± 1.76	0.97 ± 1.65	-0.23 ± 1.69	0.034
CLIUI	-1.46 ± 3.14	-4.14 ± 3.43	0.16 ± 1.40	< 0.001
CLIIP	-1.23 ± 2.54	-3.17 ± 2.65	-0.07 ± 1.63	< 0.001
CUIGIP	0.15 ± 1.63	0.89 ± 1.40	-0.30 ± 1.62	0.024
CLIGUIG	-1.61 ± 3.81	-4.74 ± 4.21	0.26 ± 1.87	< 0.001
CLIGIP	-1.40 ± 3.15	-3.85 ± 3.61	0.07 ± 1.59	< 0.001

*CLIGIP* cant of the lower incisal gingival margin to the inter-pupillary line, *CLIIP* cant of the lower incisal plane to the inter-pupillary line, *CLIGUIG* cant of the lower incisal gingival margin to the upper incisal gingival margin, *CLIUI* cant of the lower incisal plane to the upper incisal plane, *CUIGIP* cant of the upper incisal gingival margin to the inter-pupillary line, *CUIIP* cant of the upper incisal plane to the inter-pupillary line.

## Discussion

This study is the first to evaluate the effectiveness of an orthodontic appliance specifically for incisal plane cant correction. Based on the study results, the null hypothesis was rejected, indicating that AUBTs had a significant effect on incisal plane correction. Compared to other cant correction methods, AUBTs are more cost-effective as they don’t require purchasing specialized archwires or bending complex arches. Additionally, AUBTs are less invasive than skeletal anchorage. The study of Feagin et al.^19^ has shown that anterior bite turbos made with composite materials cause equal or less abrasion to opposing enamel compared to tooth-to-tooth contact. However, Pairatchawan et al.^20^ demonstrated that anterior bite planes can cause incisal root volume changes and should be avoided in teeth with short roots or susceptibility to root resorption.

Accurate clinical assessment of occlusal cant remains a challenge, and recent advancements, such as the development of the occlusal canting identifying tool, have demonstrated high validity and reliability in quantifying maxillary occlusal cants^21^. Besides incisal plane cant, other factors that may affect smile esthetics include variations in tooth size, midline deviation, as well as the mesiodistal angulation and torque of canines, central incisors, and molars^2,22,23^.

AUBTs possibly level the incisal plane by intruding the ipsilateral canine or lateral incisor through occlusal forces, while intermaxillary elastics extrude the contralateral canine and lateral incisor. The resulting posterior inter-arch spaces enable posterior teeth extrusion, similar to the effect of symmetric anterior bite turbos^16^. Precise measurement of intrusion and extrusion is challenging without anteroposterior cephalograms. However, the minimal change in the SNMP angle suggests that the vertical dimension was maintained after AUBT treatment, indicating that teeth were not excessively extruded, which can be detrimental to periodontal health. Moreover, the greater severity of pretreatment CLIIP and CLIGIP compared to CUIIP and CUIGIP in the primary group, as well as their post-iatrogenic exacerbation in the iatrogenic group, suggests that the lower incisal plane and gingival margin play a more significant role in the cant of the lower to upper incisal plane and asymmetric overbite. These findings indicate that correction of lower incisal discrepancies requires more substantial intervention. Additionally, the more pronounced improvements in CLIIP and CLIGIP compared to their upper counterparts further confirm that AUBTs exert a stronger corrective effect on the lower incisal plane and gingival margin cants than on the upper incisal plane. These findings suggest that the effect of AUBTs is primarily the intrusion of the extruded canine and lateral incisor, particularly in the lower arch, on the side with a deeper overbite.

Among the causes of iatrogenic incisal plane cant, unilateral distalization was the most common. In all cases, this procedure was performed on the lower arch and anchored with mini-screws placed at the mandibular buccal shelf. It aimed to correct lower midline deviations and Class III dental relationships on one side, which can occur during both extraction and non-extraction treatment. However, the distalizing forces can rotate the lower arch counterclockwise, leading to extrusion of the lower incisors on the distalized side and a subsequent incisal plane cant.

Additionally, asymmetric intermaxillary elastics, such as cross elastics or unilateral anteroposterior elastics, can contribute to incisal plane cant^24^. Different vertical bracket positions between the left and right sides may also cause incisal plane cant. In the case of cant due to displaced teeth, a blocked-out lower left second premolar intruded the lower left anterior teeth after the leveling stage. In one of the 4 incisal plane cases during space closure, an asymmetric extraction pattern was utilized, in which the lower left first premolar and right second molar were extracted. The resulting difference in anchorage level might have led to more anterior retraction and possibly more bowing effect on the left side, causing the cant. However, the mechanics behind the cant in the remaining three space closure cases with symmetric extraction patterns remain unclear and may be attributed to clinician-related factors, such as differences in bracket positioning between the left and right sides, or patient-related factors, such as asymmetric chewing patterns.

The immediate difficulties in chewing after AUBT placement are consistent with the reduced masticatory muscle activity in the study of Wasinwasukul et al.^25^. However, the muscle activity returned to baseline values after 1–3 months in that study, which aligns with the present study results. The initial reduction in muscle activity may be explained by the disocclusion of posterior teeth, while the subsequent recovery of muscle activity could be attributed to the re-establishment of posterior occlusion^25^. The ABUT failure rate is comparable to the lingual bracket failure rate in the study of Tepedino et al.^26^.

In the primary group, the lack of significant change in all cant parameters at T1 suggests that the pretreatment incisal plane cant was not significantly improved after the leveling stage. This finding indicates that stiff stainless steel continuous archwires may not be able to fully level the occlusal plane, likely due to their inherent flexibility. This observation aligns with other studies showing that continuous archwires cannot completely level the curves of Spee^27,28^. In contrast, the iatrogenic group showed a significant worsening of the relative cant between the lower and upper incisal planes during treatment. This suggests that improper asymmetric force systems may cause significant incisal plane cant between upper and lower arches.

The significant improvement in all cant parameters after AUBT placement, both compared to pre-AUBT levels in both groups and to pretreatment levels in the primary group, supports the effectiveness of AUBTs in reducing incisal plane cant in both the upper and lower arches. The lack of significant differences in all cant parameters between pretreatment (T0) and posttreatment (T2) in the iatrogenic group suggests that AUBTs effectively reversed treatment-induced cant to its original pretreatment state. Furthermore, the absence of significant differences in cant parameter changes between pre- and post-AUBT correction across groups indicates that AUBTs exert a comparable cant correction effect on both primary and iatrogenic cants. However, the greater overall improvement in cant parameters from pretreatment to posttreatment in the primary group, compared to the iatrogenic group, may be attributed to the combined effects of the leveling phase using stiff stainless steel archwires and the AUBT correction, leading to a more pronounced correction of pre-existing cants.

Due to its minimally invasive nature, AUBTs should be the first-line approach for managing mild to moderate dental occlusal cant, especially during the finishing stage. While meticulous wire bending and intermaxillary elastics at this phase are generally aimed at achieving optimal results, they may sometimes lead to a slightly uneven overbite, which can be a concern for detail-oriented patients. AUBTs offer a simple yet effective solution, allowing for precise overbite correction on the side with greater vertical overlap in a quick, non-invasive manner, ensuring better patient acceptance. For more severe cases, where the cant is skeletal in origin or exceeds the capacity of AUBTs alone, a combined approach using AUBTs and skeletal anchorage can be considered.

This study has several limitations that should be considered. The relatively small sample size, with a predominance of female patients, may limit the generalizability of the findings to a broader population, including male patients and different ethnic backgrounds. The absence of a control group, such as one utilizing intrusion arches, rollercoaster archwires, or skeletal anchorage, prevents definitive conclusions regarding the independent effect of AUBTs on cant correction. Additionally, the follow-up period was limited to the active treatment phase, with no assessment of long-term stability post-treatment. Measurement variability remains a concern, as cant angles were determined using digital photographs and virtual protractors, which, despite standardized protocols, may introduce minor errors. Furthermore, no molds or preformed bite turbos were used, and AUBT placement varied among participants based on individual occlusal conditions, which may have affected homogeneity. Another limitation was the lack of posteroanterior cephalogram measurements, which were omitted due to the risk of increased radiation exposure. Furthermore, while AUBTs demonstrated effectiveness in addressing mild to moderate dental cants, their applicability in severe skeletal cases remains uncertain, necessitating further investigation into combined approaches with skeletal anchorage.

## Conclusions

This study demonstrates that anterior unilateral bite turbos (AUBTs) are an effective, minimally invasive approach for correcting incisal plane cant and asymmetric overbite in orthodontic patients. Primary cant can be significantly improved using AUBTs, while iatrogenic relative cant between the upper and lower incisal planes can be effectively reversed to pretreatment values. AUBTs exhibit a greater canting corrective effect on the lower incisal plane compared to the upper one, with the mandibular plane angle well-maintained after treatment. Given their simplicity, cost-effectiveness, and minimal invasiveness, AUBTs should be considered as a first-line approach for managing mild to moderate incisal plane cants. Further studies with larger sample sizes, long-term follow-up, and control groups utilizing alternative cant correction methods are necessary to validate these findings and optimize clinical protocols.

## Electronic supplementary material

Below is the link to the electronic supplementary material.

Supplementary Material 1

## Data Availability

All data generated or analysed during this study are included in this published article and its Supplementary Information file (Dataset.xlsx).
